# Identification and validation of an individualized metabolic prognostic signature for predicting the biochemical recurrence of prostate cancer based on the immune microenvironment

**DOI:** 10.1186/s40001-024-01672-3

**Published:** 2024-01-31

**Authors:** Bintao Hu, Xi Zhang, Shiqing Zhu, Chengwei Wang, Zhiyao Deng, Tao Wang, Yue Wu

**Affiliations:** 1 Department of Urology, Tongji Hospital, Tongji Medical College, Huazhong University of Science and Technology, Wuhan, Hubei China; 2 Shenzhen Huazhong University of Science and Technology Research Institute, Shenzhen, Guangdong China; 3 School of Nursing, Tongji Medical College, Huazhong University of Science and Technology, Wuhan, Hubei China

**Keywords:** Prostate cancer, Immune microenvironment, Biochemical recurrence, Immune gene, Metabolic genes, Prognostic signature

## Abstract

**Background:**

Prostate cancer (PCa) is the most prevalent genitourinary malignancy in men, with a significant proportion of patients developing biochemical recurrence (BCR) after treatment. The immune microenvironment and metabolic alterations have crucial implications for the tumorigenesis and progression of PCa. Therefore, identifying metabolic genes associated with the immune microenvironment holds promise for predicting BCR and improving PCa prognosis.

**Methods:**

In this study, ssGSEA and hierarchical clustering analysis were first conducted to evaluate and group PCa samples, followed by the use of the ESTIMATE and CIBERSORT algorithms to characterize the immunophenotypes and tumor microenvironment. The differential metabolic genes (MTGs) between groups were utilized to develop a prognostic-related signature. The predictive performance of the signature was assessed by principal component analysis (PCA), receiver operating characteristic (ROC) curve analysis, survival analysis, and the TIDE algorithm. A miRNA-MTGs regulatory network and predictive nomogram were constructed. Moreover, the expression of prognostic MTGs in PCa was detected by RT‒qPCR.

**Results:**

PCa samples from the TCGA cohort were separated into two groups: the immune-low group and immune-high group. Forty-eight differentially expressed MTGs between the groups were identified, including 37 up-regulated and 11 down-regulated MTGs. Subsequently, *CEL*, *CYP3A4*, and *PDE6G* were identified as the genes most strongly associated with the BCR of PCa patients and these genes were utilized to establish the MTGs-based prognostic signatures. PCA, ROC curves analysis, Kaplan–Meier survival analysis, and the nomogram all showed the good predictive ability of the signature regardless of clinical variables. Furthermore, the MTGs-based signature was indicated as a potential predictive biomarker for immunotherapy response. Nine miRNAs involved in the regulation of prognostic MTGs were determined. In addition to the *CEL* gene, the *PDE6G* and *CYP3A4* genes were expressed at higher levels in PCa samples.

**Conclusions:**

The MTGs-based signature represents a novel approach with promising potential for predicting BCR in PCa patients.

**Supplementary Information:**

The online version contains supplementary material available at 10.1186/s40001-024-01672-3.

## Introduction

The tumor microenvironment (TME) is a complex noncancerous component located adjacent to tumor tissue that is mainly composed of cellular and acellular parts with different proportions and components [1]. The cell components mainly included various tumor-infiltrating lymphocytes (TILs), endothelial cells, and fibroblasts [2]. The acellular components mainly consist of extracellular matrix [1]. The heterogeneous cell types within the TME can either metabolically cooperate or compete for limited nutrients [3]. Additionally, their blood vessels are often limited or poorly differentiated, leading to inefficient nutrient and oxygen transport, as well as waste removal [4]. However, an important characteristic of tumor cells compared with normal cells is their high metabolic plasticity. In such cases, the tumor metabolic microenvironment, which is an immunosuppressive environment, allows them to adapt more quickly and better to fewer nutrients or changing nutritional conditions [5]. Hensley et al. [6] found that the energy utilization patterns of non-small cell lung cancer cells were heterogeneous. Specifically, cancer cells in regions rich in vascularization could utilize energy sources other than glucose, while the cancer cells in regions with low perfusion mainly used glucose. Meanwhile, under such adverse conditions in the TME, infiltrating immune cells also undergo metabolic changes associated with immune tolerance, ultimately undermining the effectiveness of the antitumor immune response [4]. Therefore, identifying novel communication mechanisms within the TME that depend on tumor metabolic activity is crucial. Specifically, identifying targets that inhibit or alter tumor metabolism to improve the availability of nutrients in the TME or regulate immune metabolism to promote the inflammatory response, will enhance our understanding of tumorigenesis and reveal potential therapeutic targets.

Prostate cancer (PCa) is the most prevalent genitourinary tumor among men, with approximately 220,000 cases reported annually in the United States [7]. Treatment options for low-risk and localized PCa patients mainly include active surveillance, radical prostatectomy, and radiation therapy, while for higher-risk patients, adjuvant androgen deprivation therapy (ADT) is often required [8]. However, approximately 25% of patients will develop biochemical recurrence (BCR) after these treatments [9]. ADT is currently the standard treatment for patients with recurrent or metastatic prostate cancer, aiming to eliminate circulating androgens that fuel prostate cancer growth; however, most patients eventually develop castration-resistant prostate cancer, which is associated with a worse prognosis [7, 10, 11]. Hence, new treatment strategies are urgently needed.

Although immunotherapy has shown promise in various solid tumors, its efficacy in treating PCa has been limited [12]. Immune responses, including multiple cytokines in the TME, can significantly influence the balance between tumor progression and therapeutic response. To understand the dynamic changes in immune responses, it is important to consider the characteristics of the tumor immune microenvironment (TIME), such as the number of immunosuppressive cells and the infiltration of TILs [13]. The TME of PCa exhibits unique characteristics [14]. For example, high CD8^+^ TIL infiltration, especially activated TIL infiltration, has been associated with improved prognosis in patients with various solid tumors [15]. However, the relationship between CD8^+^ TIL infiltration and the prognosis in PCa patients remains unclear. Several studies have shown that high TIL infiltration is associated with shorter BCR time and poorer clinical outcomes in patients with PCa [16]. In addition, a high proportion of CD4^+^ and CD8^+^ regulatory T cells (Tregs) expressing forkhead box P3 (Foxp3^+^) have been observed in the margins and epithelial compartments of PCa tumors [17]. Thus, an in-depth understanding of the TIME characteristics in PCa, especially changes in tumor metabolism in the TIME, will contribute to the development of new therapeutic strategies. Herein, we classified PCa patients from The Cancer Genome Atlas (TCGA) cohort based on their immunophenotype and further investigated the changes in metabolic activity, metabolism-related molecular characteristics, and the prognostic potential of these characteristics.

## Materials and methods

### PCa data acquisition and preprocessing

We downloaded RNA sequencing data (read counts), miRNA sequencing data, somatic mutation data (MAF values), and clinical data containing BCR information of PCa patients from the TCGA database (https://portal.gdc.cancer.gov/). The “edgeR” package was utilized to process the read count data, which included taking the average of genes with the same gene symbol, excluding genes whose average expression level was less than 1, and performing normalization. Next, to find another cohort as an external cohort to validate, we downloaded the GSE70769 cohort (*n* = 94) from the Gene Expression Omnibus (GEO) database (http://www.ncbi.nlm.nih.gov/geo/). Subsequently, the robust multiarray analysis (RMA) algorithm was applied to the original microarray data for background adjustment and normalization.

### Single-sample gene set enrichment analysis (ssGSEA) and hierarchical clustering analysis

SsGSEA is a method for comprehensively assessing the relative number of immune cells in different samples [18]. In this study, the immune characteristics of each PCa sample from the TCGA cohort were comprehensively evaluated by the 29 immune gene sets provided in the ssGSEA algorithm. Then, following a cluster analysis based on Ward's linkage and Euclidean distance, PCa patients were grouped into low-immunity and high-immunity groups.

### Cell type identification by estimating relative subsets of RNA transcripts (CIBERSORT)

The deconvolution algorithm CIBERSORT was used to assess the quantity of infiltrating immune cells in each sample [19]. Here, we employed this algorithm utilizing 22 gene sets to evaluate the extent of immune cell infiltration in different PCa groups. The parameters were set to 1000 permutations, and *P* < 0.05 was the screening standard.

### Estimation of the PCa immune microenvironment

The estimation of stromal and immune cells in malignant tumor tissues using expression data (ESTIMATE) is a sophisticated method for assessing the extent of tumor and normal cell infiltration in each sample [20]. In this study, we evaluated the tumor microenvironment of PCa samples by using this algorithm and determined the stromal score, immune score, and estimate score.

### Identification of differentially expressed metabolic genes (MTGs)

Seventy metabolism-related gene sets were obtained from a subset of 186 gene sets included in the Kyoto Encyclopedia of Genes and Genomes (KEGG) subset of canonical pathways via the Gene Set Enrichment Analysis (GSEA) website (https://www.gsea-msigdb.org/gsea/msigdb/genesets.jsp?collection=CP:KEGG). Moreover, the final expression data were then obtained based on the TCGA cohort. Subsequently, the differentially expressed MTGs between the low-immunity and high-immunity groups were identified by the “edgeR” package. In this study, the selection criteria for differentially expressed MTGs were as follows: |log_2_ fold change (FC)|> 1 and a false discovery rate (FDR) < 0.05.

### MTGs-based prognostic-related signature

The training set (*n* = 206) and test set (*n* = 206) were created randomly from the entire TCGA cohort. The training set was used to establish the MTGs-based prognostic-related signature, while the entire and test sets were used to verify the established signature. The potential prognostic value of these MTGs was revealed by univariate Cox regression analysis. Subsequently, we performed least absolute shrinkage and selection operator (LASSO) and multivariate Cox regression analyses to identify the MTGs most strongly associated with prognosis. Finally, we calculated the risk score of the prognostic signature using the following formula with the β coefficient of Cox regression analysis and the corresponding MTGs expression value:

$$Risk\,score\, = \,\sum\nolimits_{i = 1}^{n} {Expi\beta i}$$

β and Exp in the above formula represent the regression coefficients and expression values of the corresponding genes, respectively. Following the calculation of each patient’s risk score, PCa patients in the training set were split into two groups (the high-risk and low-risk groups) on the basis of the median risk score. The variations in BCR between the two groups were compared using Kaplan‒Meier survival analysis. The predictive power of the MTGs-based prognostic signature was assessed by the area under the ROC curves (AUCs) and the receiver operating characteristic (ROC) curves. Furthermore, the test set, the entire TCGA cohort, and the GSE70769 cohort were used to validate the stability and reliability of the prognostic signature.

### Analysis of the prognostic signature and prognostic MTGs stratified by different clinical variables

To reveal the prognostic significance of the signature among PCa patients under different clinical stratifications, we carried out Kaplan‒Meier survival analysis. The expression levels of the MTGs under stratification were also compared with the aim to preliminarily reveal the possible role of these MTGs in PCa.

### Evaluation of the grouping ability of the signature by principal component analysis (PCA)

Based on the expression patterns of the MTGs-based prognostic-related signatures, principal component analysis (PCA) was employed to effectively decrease the number of dimensions, identify the signature, and visualize the high-dimensional data of the training set gene expression profile, the test set gene expression profile, all MTGs, and the risk signatures.

### miRNA-MTGs regulatory network and functional enrichment analysis

After preprocessing and differential analysis of the miRNA expression data, we performed a coexpression analysis of the prognosis-related MTGs and miRNAs. The criteria for relevance were |Cor|> 0.3 and *P* < 0.001. Subsequently, to elucidate the related biological functions and molecular pathways, Kyoto Encyclopedia of Genes and Genomes (KEGG) enrichment analysis and Gene Ontology (GO) analysis were conducted on these differentially expressed MTGs by utilizing the “clusterProfiler” package.

### The potential of the signature to serve as a measure of immunotherapy response in PCa patients

The expression patterns of immune checkpoint inhibitor (ICI) genes (*CTLA-4*, *PD-L1*, *PD1*, *PD-L2*, *B7H3*, and *B7H4*) in various patient groups were compared based on signature stratification. Further research was conducted to determine whether the signature affects the clinical outcomes of patients with comparable immune checkpoint gene expression levels. In the absence of data on PCa patients receiving immunotherapy, the predictive performance of the tumor response to immunotherapy was assessed using the TIDE algorithm created by Jiang et al. [21]; this algorithm was applied to preliminarily evaluate the response rate of PCa patients to immunotherapy under signature stratification. Moreover, we utilized the “maftools” package to evaluate the mutation rate and mutation load in PCa patients stratified according to the signature.

### Construction and assessment of a predictive nomogram

We conducted Cox regression analyses to determine whether this MTGs-based prognostic signature was associated with BCR when considering other clinical variables in PCa patients (such as T stage, N stage, Gleason score, and age). A nomogram containing clinical variables and the risk score was then developed through the “rms” R package. Furthermore, we evaluated the accuracy of the nomogram (assessing the degree of deviation between the predicted and actual values) by calibration curve analysis. Moreover, the predictive performance and stability of the nomogram were assessed utilizing the TCGA and GSE70769 cohorts.

### Analysis of the expression of prognostic MTGs in PCa

PCa transcriptome data from the TCGA cohort were utilized to compare differences in the expression of prognostic MTGs between tumor and normal tissues. With regard to gene expression in tumor cells, the expression of prognostic MTGs in various PCa cell lines was analyzed by the use of Cancer Cell Line Encyclopedia (CCLE) database. Furthermore, total RNA was extracted from PCa cell samples using a SteadyPure RNA Extraction Kit (without Lysis Buffer) (Accurate Biotechnology, Hunan, China) and reverse transcribed into cDNA, followed by cDNA amplification and qPCR detection (Yeasen, Shanghai, China). The primer sequences for the prognostic MTGs are provided in Additional file 2: Table S2.

## Results

### Immunophenotype and tumor microenvironment of PCa patients in the TCGA cohort

**Fig. 1:**
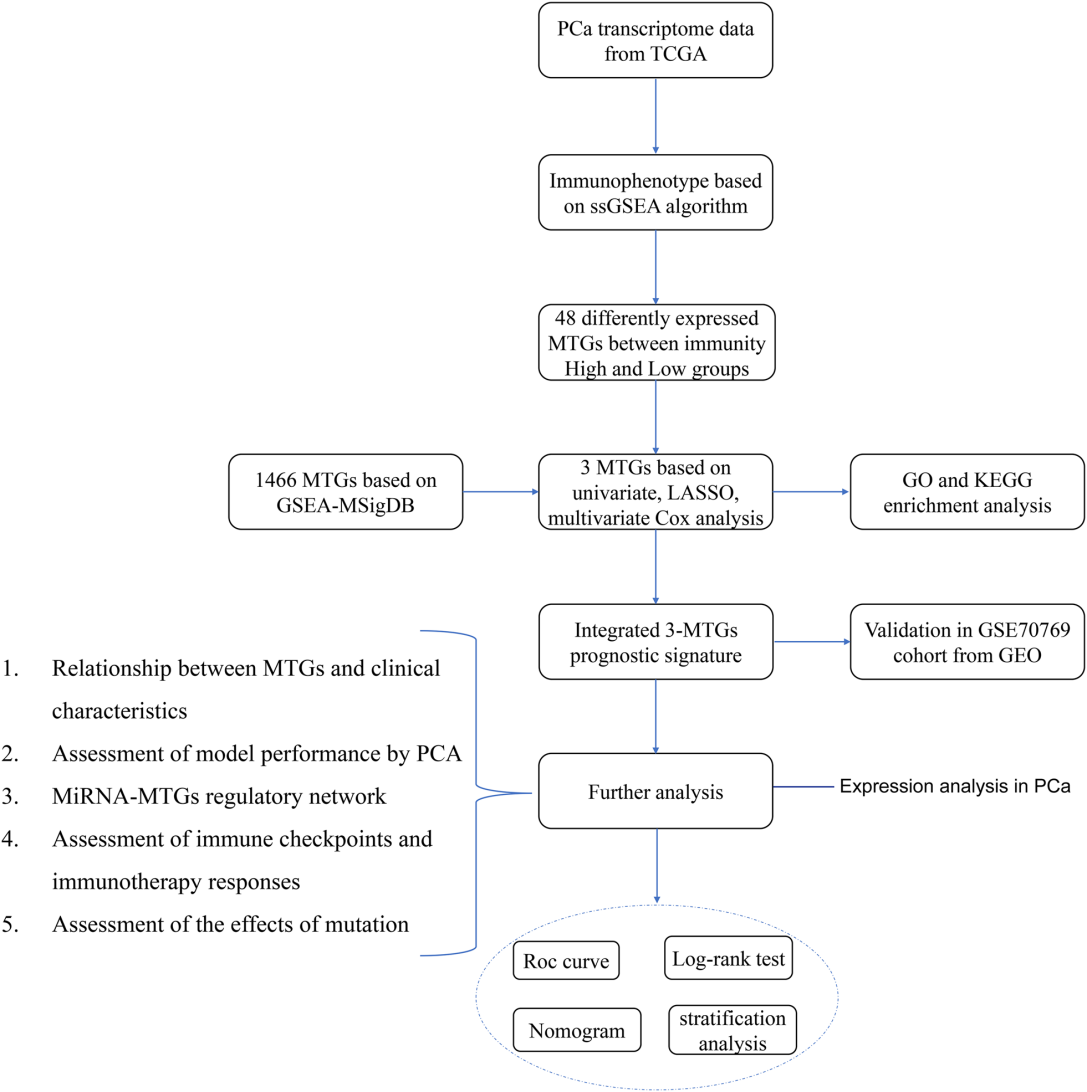
Flowchart of the prognostic signature for PCa

**Fig. 2:**
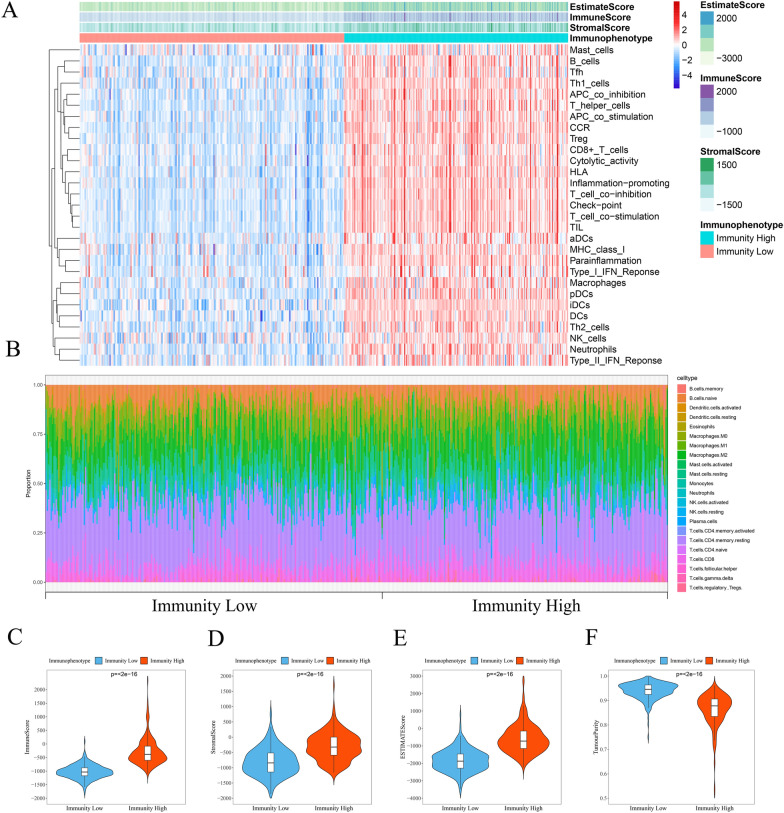
The tumor microenvironment and immunophenotype of patients with PCa in the TCGA cohort. **A** The ssGSEA algorithm and hierarchical cluster analysis were used to determine the immune characteristics and tumor microenvironment landscape of PCa patients. **B** Differences in the infiltration of 22 immune cells between the low-immunity and high-immunity groups were determined based on the CIBERSORT algorithm. The violin plot displays the ImmuneScore (**C**), StromalScore (**D**), EstimateScore (**E**), TumourPurity (**F**) between the low-immunity and high-immunity groups

Figure 1 presents an overview of this study design. The immune characteristics (including the activity or enrichment of immune cells, function, pathway, or checkpoint) of 489 PCa samples in the TCGA cohort were comprehensively evaluated via the ssGSEA algorithm. After hierarchical clustering, the PCa samples were divided into two categories: the low-immunity group containing 265 samples with low immune characteristics, and the high-immunity group containing 224 samples with high immune characteristics (Fig. 2A). Subsequently, we utilized the ESTIMATE algorithm to score the TME of each sample in the TCGA cohort and compared the TME characteristics between the two groups. The StromalScore, ImmuneScore, and ESTIMATEScore in the high-immunity group were −308.232 ± 446.419, −292.195 ± 486.932, and −600.427 ± 807.535, respectively. While the StromalScore, ImmuneScore, and ESTIMATEScore in the low-immunity group were −807.462 ± 446.845, −1025.709 ± 250.929, and −1833.171 ± 635.311, respectively. Compared to the low-immunity group, the high-immunity group exhibited a significant difference in scores, with higher levels of stromal cells and immune cell infiltration (Fig. 2C–F). Furthermore, the CIBERSORT algorithm showed the difference between the high-immunity group and low-immunity group in immune cell infiltration (Fig. 2B). Specifically, the infiltration levels of regulatory Tregs, CD4 memory activated T cells, CD4 memory resting T cells, CD8 T cells, plasma cells, memory B cells, naive B cells, activated dendritic cells, resting dendritic cells, M2 macrophages, monocytes, activated NK cells, resting NK cells, and resting mast cells exhibited significantly different between the two groups. These findings indicated the successful classification of PCa patients based on their immunophenotype.

### Differentially expressed MTGs analysis

**Fig. 3:**
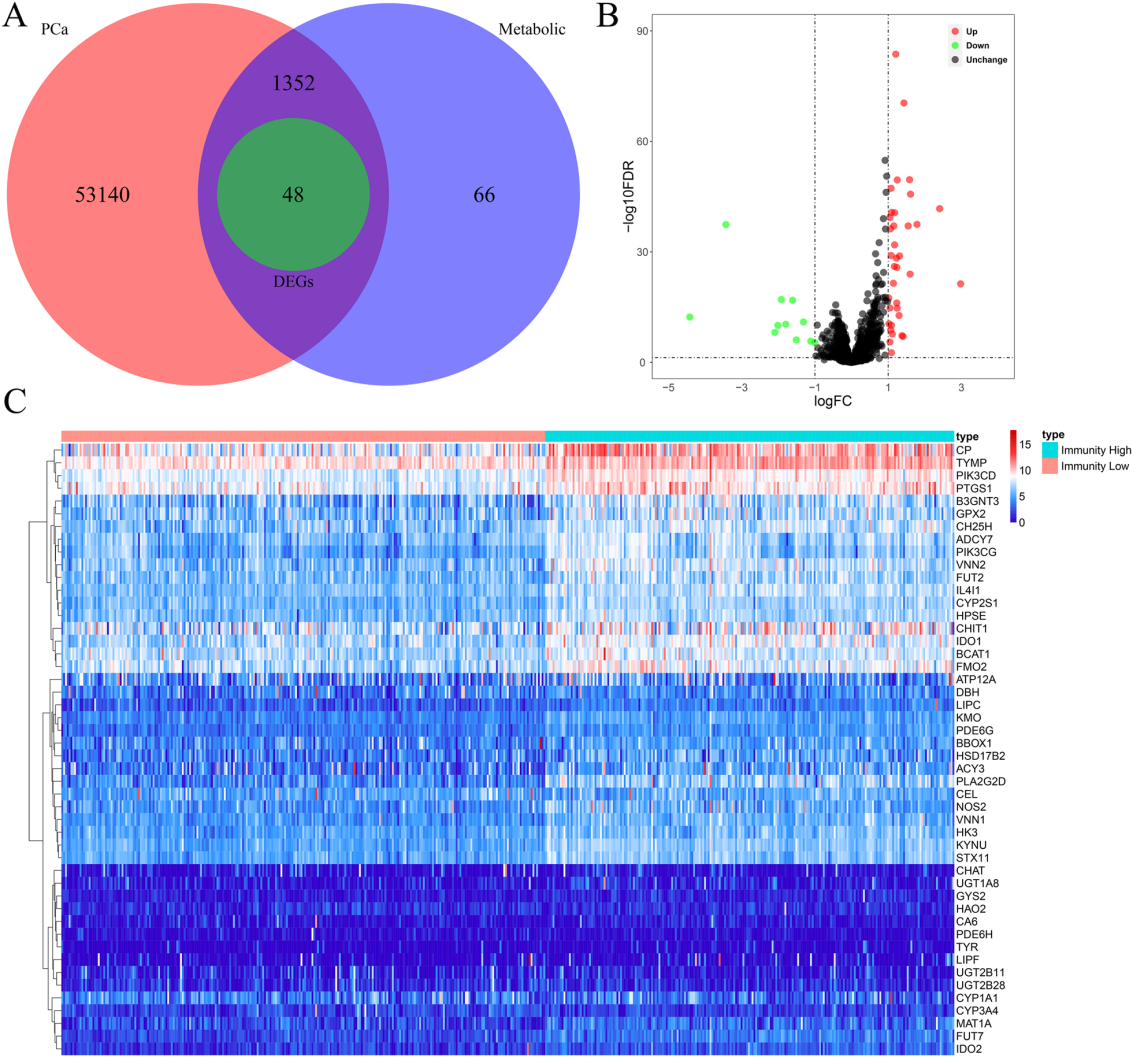
Analysis of differentially expressed MTGs between the low-immunity and high-immunity groups. **A** Venn diagram of intersecting genes between PCa and metabolism based on immunophenotype. **B** Volcano plot of differentially expressed MTGs. Red represents upregulated MTGs and green represents downregulated MTGs. **C** Heatmap of 48 differentially expressed MTGs

After classifying PCa patients into high-immunity/low-immunity groups, we further investigated the variations in metabolic activity between the two groups. We first identified 1466 MTGs from 70 KEGG metabolic gene sets and then obtained the expression data of 1400 MTGs based on the TCGA cohort (Fig. 3A). Following data analysis, we identified 48 MTGs with differential expression between the low- and high-immunization groups, including 11 downregulated and 37 upregulated MTGs. Figure 3B, C display a volcano plot of the 1400 MTGs and a heatmap of 48 MTGs, respectively.

### Establishment and assessment of an MTGs-based prognostic signature

The prognostic significance of the differentially expressed MTGs in PCa patients was investigated. Test (*n* = 206) and training (*n* = 206) groups of PCa patients from the entire TCGA cohort were created using the “caret” R package. Subsequently, we performed univariate Cox regression analysis for the MTGs with differential expression, revealing that 5 MTGs, *CA6*, *CEL*, *CYP3A4*, *KYNU*, and *PDE6G*, were associated with BCR. Additionally, LASSO regression analysis was conducted on the above 5 MTGs through “glmnet” package for further filtering. Additional file 1: Figure S1A presents the trajectory changes in these 5 MTGs according to LASSO regression analysis. Additional file 1: Figure S1B shows the model built by using the cross-validation method. All 5 of these MTGs were proved to be closely related to BCR in PCa. Afterward, further multivariate Cox regression analysis identified the three most prognostically relevant MTGs: *CEL*, *CYP3A4*, and *PDE6G* (Table 1).

**Table 1:** Multivariate Cox regression analysis to identify prognosis-related metabolic genes

Gene	Coef*	Exp(coef)	se(coef)	z	Pr( >|*z*|)
*CEL*	0.1033	1.1088	0.1023	1.0096	0.3127
*CYP3A4*	0.2630	1.3008	0.1181	2.2278	0.0259
*PDE6G*	0.3849	1.4695	0.1963	1.9613	0.0498

^*^Coef: coefficient

We calculated the risk score for the prognostic signature with the use of the β coefficient from Cox regression analysis and the corresponding MTGs expression values, according to the formula below:

Risk score = (0.1033*Exp *CEL*) + (0.2630*Exp *CYP3A4*) + (0.3849*Exp *PDE6G*).

**Fig. 4:**
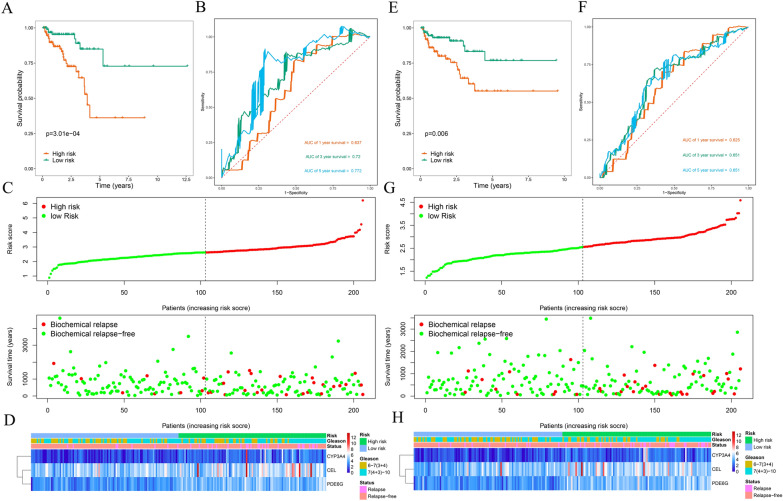
Risk score analysis of the prognostic signature based on training and test subsets. **A** Kaplan–Meier survival curve analysis of BCRs between the high- and low-risk groups in the training subset. **B** 1-year, 3-year, and 5-year ROC curves of PCa patients in the training subset. **C** Risk score and survival status of each PCa patient in the training subset. **D** Heatmap of prognostic MTGs expression based on the risk score and clinical characteristics in the training subset. **E** Kaplan–Meier survival curve analysis of BCR between the high- and low-risk groups in the test subset. **F** 1-year, 3-year, and 5-year ROC curves of PCa patients in the test subset. **G** Risk score and survival status of each PCa patient in the test subset. **H** Heatmap of prognostic MTGs expression based on the risk score and clinical characteristics of the test subset

**Fig. 5:**
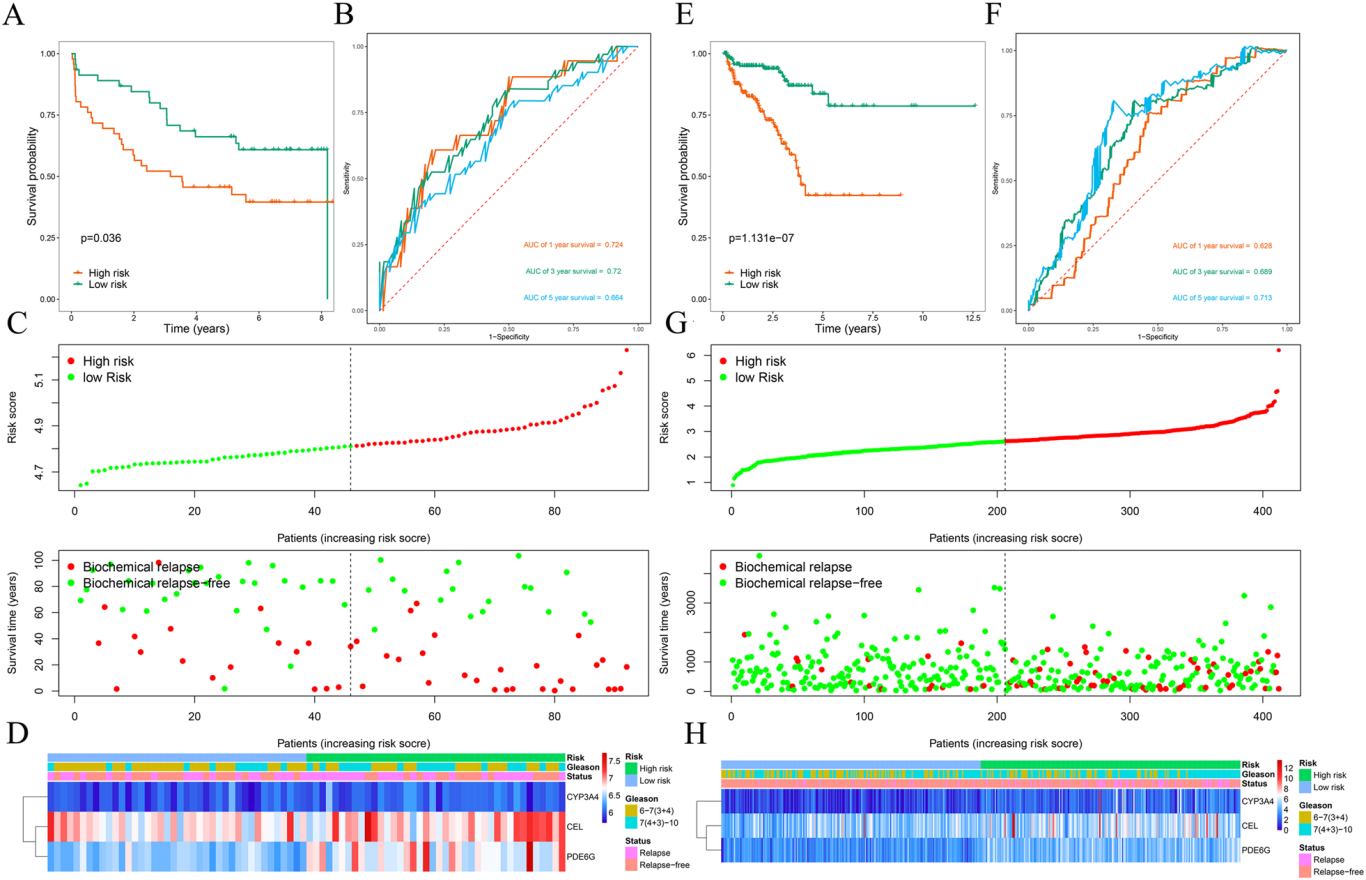
Risk score analysis of the prognostic signature based on the GSE70769 and entire TCGA cohorts. **A** Kaplan–Meier survival curve analysis of BCRs between the high- and low-risk groups in the GSE70769 cohort. **B** 1-year, 3-year, and 5-year ROC curves of PCa patients in the GSE70769 cohort. **C** Risk score and survival status of each PCa patient in the GSE70769 cohort. **D** Heatmap of prognostic MTGs expression based on the risk score and clinical characteristics in the GSE70769 cohort. **E** Kaplan–Meier survival curve analysis of BCRs between the high- and low-risk groups in the entire TCGA cohort. **F** 1-year, 3-year, and 5-year ROC curves of PCa patients in the entire TCGA cohort. **G** Risk score and survival status of each PCa patient in the entire TCGA cohort. **H** Heatmap of prognostic MTGs expression based on the risk score and clinical characteristics in the entire TCGA cohort

Then, a low-risk group (*n* = 103) and a high-risk group (*n* = 103) were generated in the training group based on the calculated median risk score. PCa patients in the high-risk group showed a worse prognosis than did those in the low-risk group, as indicated by survival analysis (*P* = 3.01e-04; Fig. 4A). ROC curve analysis was then performed to evaluate the predictive performance of the MTGs-based signature, and the area under the curve (AUC) for BCR at 1 year, 3 years, and 5 years were 0.637, 0.720, and 0.772, respectively (Fig. 4B). Figure 4C shows the BCR distribution of PCa patients assessed by the risk score, and Fig. 4D displays a heatmap of the expression of the three prognostic MTGs. Furthermore, the same formula was applied to calculate risk scores in the test group (Fig. 4E–H), the GSE70769 cohort (Fig. 5A–D), and the entire TCGA cohort (Fig. 5E–H). The predictive performance of the signature was assessed by survival and ROC curve analysis, and similar results were observed across the different cohorts. These results demonstrated that our MTGs-based signature had high predictive performance and could accurately distinguish PCa patients at risk of BCR.

### Stratified analysis of the prognostic signature and prognostic MTGs

**Fig. 6:**
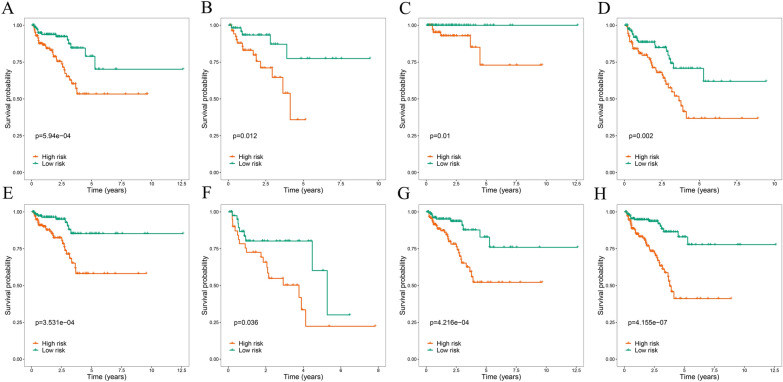
Kaplan–Meier survival curve analysis of BCRs stratified by different clinical parameters. **A** Age ≤ 65. **B** Age > 65. **C** Gleason score 6–7 (3 + 4). **D** Gleason score 7(4 + 3)-10. **E** T stage 1–2. **F** T stage3-4. **G** N stage0. **H** M stage0

To investigate whether different clinical variables affect the prognostic significance of the signature, survival analysis in PCa patients stratified by these variables was conducted. The results consistently demonstrated that PCa patients in the high-risk group presented a less favorable outcome across various stratification levels (Fig. 6). And the signature was suggested to accurately predict BCR in PCa patients regardless of other clinical variables. Furthermore, we additionally explored the potential role of these prognostic MTGs in PCa patients stratified according to different clinical variables. The expression levels of *PDE6G* and *CYP3A4* were significantly correlated with the Gleason score. Moreover, the level of *PDE6G* expression showed a significant correlation with N stage. However, no significant associations between prognostic MTGs and age or T stage were observed (Table 2).

**Table 2:** Relationships between prognosis-related metabolic genes and clinicopathologic parameters

Gene		Age		Gleason		T stage		N stage		Title 4
		≤ 65	> 65	6−7 (3 + 4)	7 (4 + 3)−10	T1-T2	T3-T4	N0	N1	
N		297	115	158	254	318	92	293	67	Data
*CEL*	*t*	0.274		1.649		0.555		0.268		Data
	*P*	0.784		0.100		0.579		0.789		Data
*PDE6G*	*t*	1.963		4.171		0.201		2.950		Data
	*P*	0.050		< 0.001		0.841		0.003		Data
*CYP3A4*	*t*	0.134		2.476		0.231		0.868		Data
	*P*	0.893		0.014		0.817		0.386		Data

### PCA analysis further revealed the grouping ability of the MTGs-based signature

**Fig. 7:**
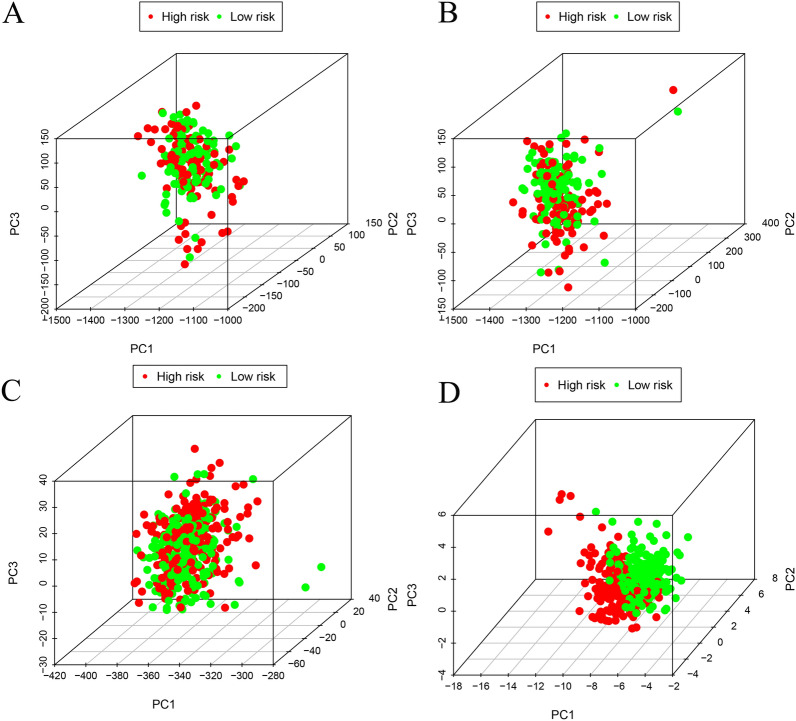
The grouping ability of the MTGs-based signature determined by PCA. **A** PCA based on the training set gene expression profile. **B** PCA based on the test set gene expression profile. **C** PCA based on all the MTGs. **D** PCA based on the risk signature

We used PCA to effectively reduce dimensions, with the aim to explore the distribution of discrepancies between the two groups of patients in the training set gene expression profile, the test set gene expression profile, all MTGs, and the risk signature. The distributions of patients in the low- and high-risk groups were relatively scattered, as presented in Fig. 7A–C. However, based on our risk signature, high/low-risk PCa patients were separated into two distinct directions (Fig. 7D). The results indicated that the model could correctly distinguish PCa patients at risk of BCR.

### miRNA-MTGs regulatory network and functional enrichment analysis

**Fig. 8:**
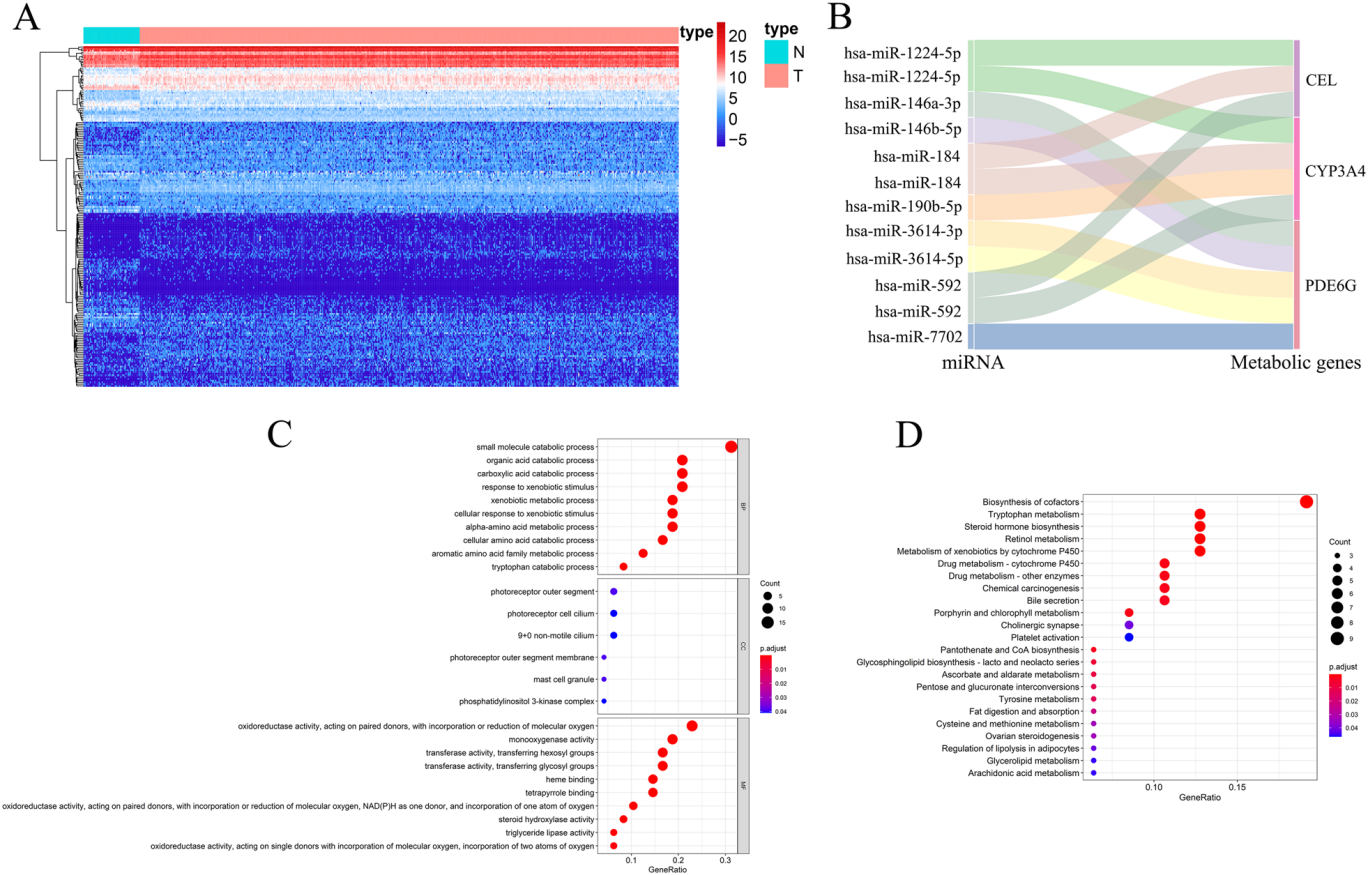
miRNA-MTGs regulatory network and functional enrichment analysis. **A** Heatmap of differentially expressed miRNAs between PCa tumor and normal tissues. Red represents the tumor group, and green represents the normal group. **B** A Sankey plot of the differentially expressed miRNAs and prognostic MTGs regulatory networks. **C** GO enrichment analysis of the differentially expressed MTGs; **D** KEGG enrichment analysis of the differentially expressed MTGs

miRNAs are small noncoding RNAs that prevent protein translation or induce posttranscriptional inhibition by targeting messenger RNA (mRNA) [22]. They have been found to be involved in the regulation of almost all cancer characteristics. Recently, the role of miRNAs in the regulation of tumor metabolism has attracted increasing attention [23]. Studies have shown that miRNAs can directly regulate the expression of metabolic transporters [24] or metabolic enzymes [25] and indirectly modulate metabolic activity by regulating the expression of major transcription factors or signaling proteins in metabolic pathways [26]. Therefore, in this study, given the important role of miRNAs in tumor metabolic reprogramming, we deeply explored the miRNA-MTGs regulatory network in PCa. We downloaded miRNA sequencing data for PCa patients from the TCGA database, which included 499 tumor samples and 52 normal samples. After differential expression analysis, 194 dysregulated miRNAs were obtained, among which 118 were upregulated and 76 were downregulated. A heatmap of the differentially expressed miRNAs is shown in Fig. 8A. Afterward, we conducted a coexpression study on the prognostic MTGs and miRNAs with differential expression and identified 9 miRNAs involved in the regulation of prognostic MTGs, namely, has-miR-1224-5p, has-miR-184, has-miR-592, has-miR-190b-5p, has-miR-146a-3p, has-miR-146b-5p, has-miR-3614-3p, has-miR-3614-5p, and has-miR-7702. Figure 8B presents the Sankey plot of the miRNA-MTGs regulatory network. All the miRNAs positively regulated the expression of MTGs, and the specific regulatory relationships are shown in Additional file 2: Table S1. The above results indicated that these miRNAs may regulate tumor growth in PCa, which is worthy of more research.

Furthermore, to explore the cellular functions and underlying mechanisms of these 48 differentially expressed MTGs, GO and KEGG enrichment analyses were conducted. Biological process analysis revealed that the differentially expressed MTGs were mainly involved in the organic acid catabolic process, cellular amino acid catabolic process, aromatic amino acid family metabolic process, and small molecule catabolic process. Regarding the cellular component, the differentially expressed MTGs were primarily associated with components such as the photoreceptor outer segment membrane, mast cell granule, and the phosphatidylinositol 3-kinase complex. The molecular function analysis revealed that the differentially expressed MTGs primarily exhibited transferase activity, transferring glycosyl groups, heme binding, and oxidoreductase activity, acting on paired donors, with incorporation or reduction of molecular oxygen (Fig. 8C). And the KEGG analysis showed that the differentially expressed MTGs were mainly enriched in pentose and glucuronate interconversions, pantothenate and CoA biosynthesis, steroid hormone biosynthesis, and tryptophan metabolism (Fig. 8D).

### The signature serves as a potential indicator of immunotherapy response in PCa patients

**Fig. 9:**
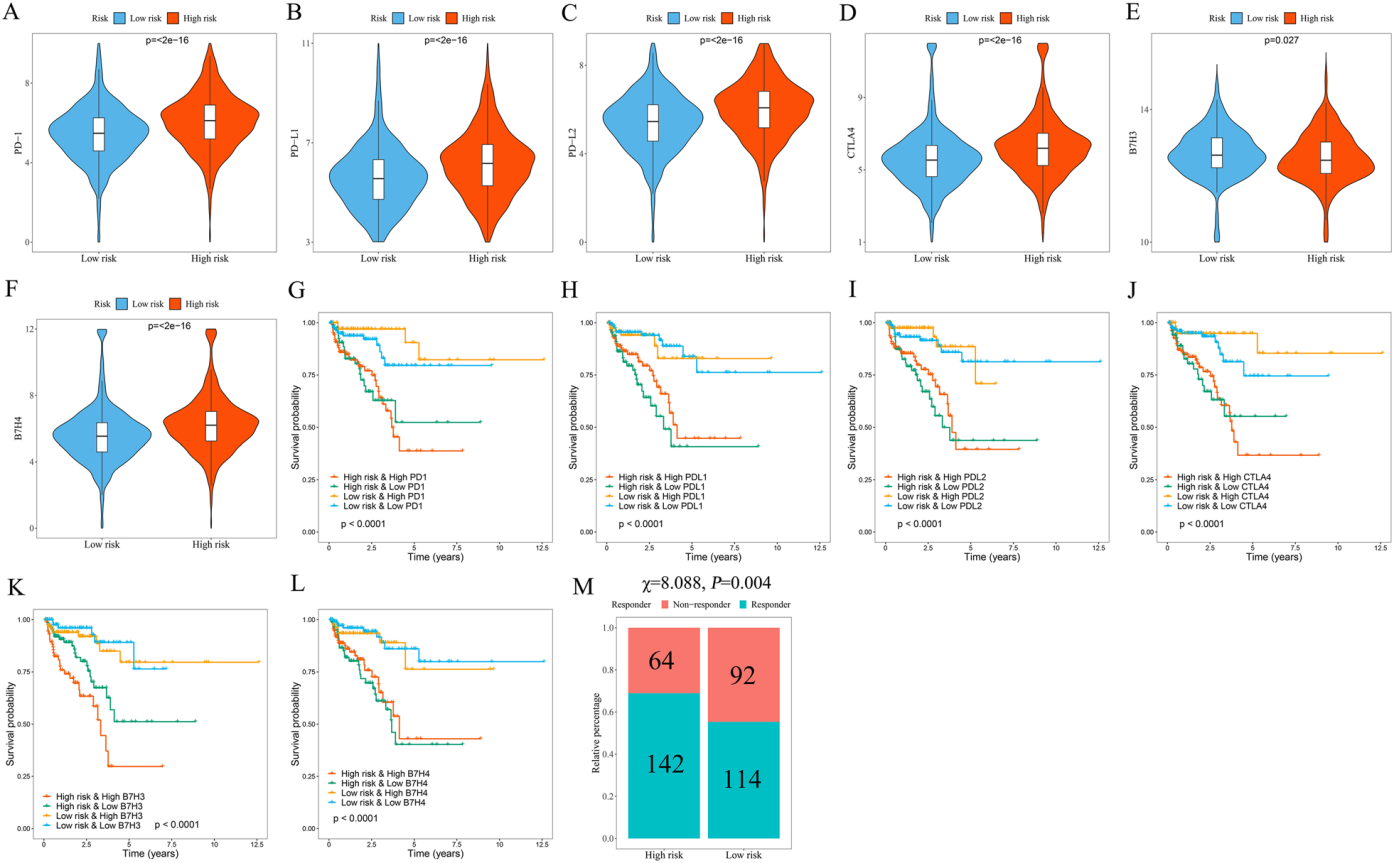
The potential of the signature as an indicator of immunotherapy response in PCa patients. Distribution of *PD-1* (**A**), *PD-L1* (**B**), *PD-L2* (**C**), *CTLA4* (**D**), *B7H3* (**E**), and *B7H4* (**F**) expression in the high and low-risk groups. Kaplan–Meier survival curves for the four patient groups stratified by risk score and *PD-1* (**G**), *PD-L1* (**H**), *PD-L2* (**I**), *CTLA4* (**J**), *B7H3* (**K**), and *B7H4* (**L**) expression. **M** The response rate to immunotherapy in the TCGA cohort of PCa patients based on the TIDE algorithm

ICI genes can modulate immune infiltration in the TME, as shown in previous studies [27]. The differences in the expression of ICI genes (*B7H3*, *B7H4, CTLA-4*, *PD-L1*, *PD-L2*, and *PD1*) among the different groups stratified by signature were compared with the aim to further explore the impact of ICI genes on MTGs in the context of immune infiltration. These ICI genes in the high-risk group were shown to be expressed at significantly higher levels (Fig. 9A–F), which is in line with earlier research demonstrating a link between adverse outcomes and high expression of ICI genes [27]. Furthermore, in the patients with comparable expression of ICI genes, the effect of the signature on clinical outcomes was investigated. Patients with high *PD-1* expression and low risk demonstrated improved outcomes compared with those with high *PD-1* expression and high risk, while patients with low *PD-1* expression and low risk showed improved outcomes compared with those with low *PD-1* expression and high risk (*P* < 0.001, Fig. 9G). Comparable results were found in other ICI genes (*P* < 0.001, Fig. 9H–L). Moreover, patients in the high-risk group exhibited lower response rates to immunotherapy, as revealed by TIDE analysis (*P* = 0.004, Fig. 9M). According to the aforementioned findings, the prognostic signature might serve as a possible predictive indicator for immunotherapy response.

**Fig. 10:**
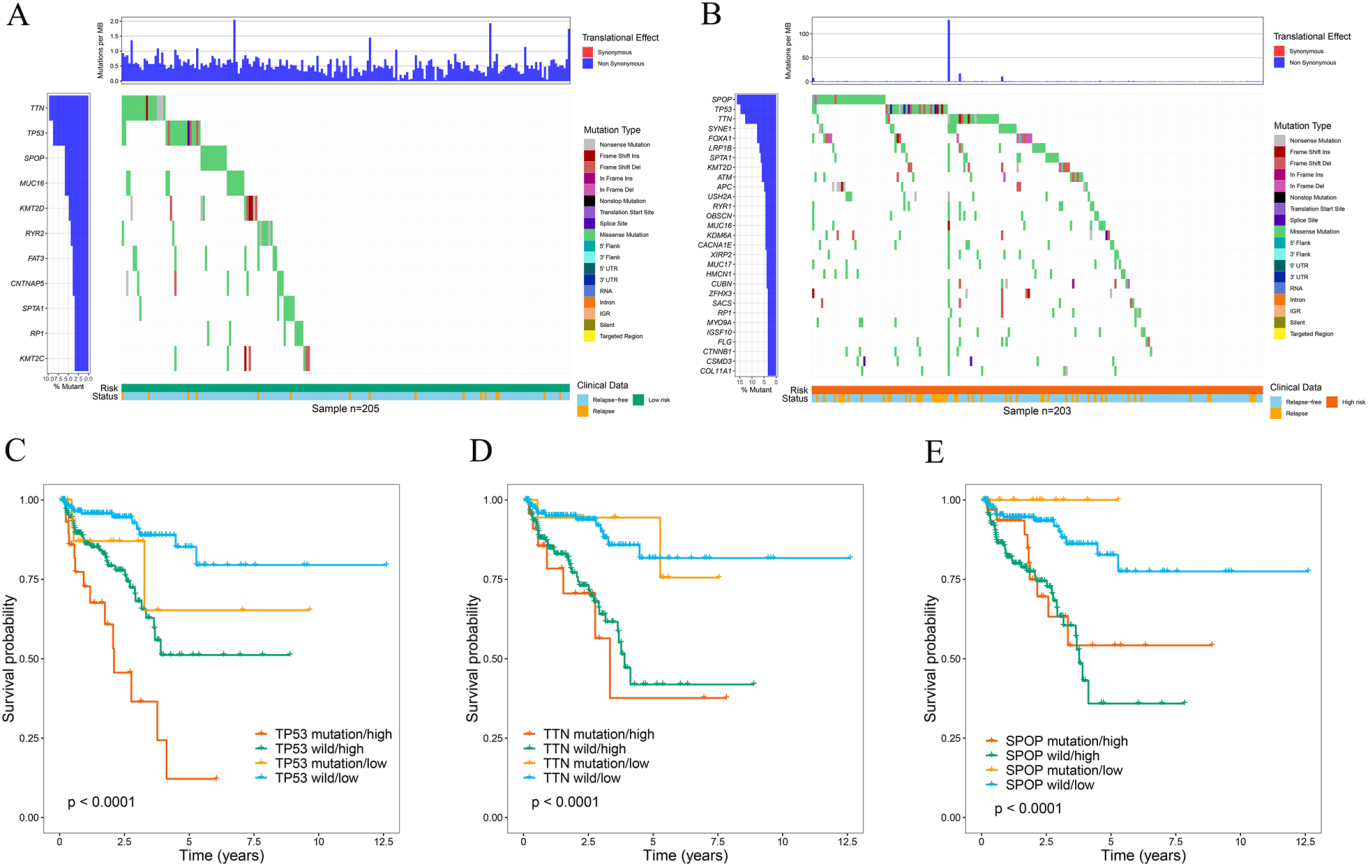
Mutation differences between high- and low-risk patients in the TCGA cohort. **A** The waterfall plot shows mutation information for genes that were mutated in at least 3% of the samples in the high-risk group. **B** The waterfall plot shows mutation information for genes that were mutated in at least 3% of the samples in the low-risk group. Kaplan–Meier survival curves for the four patient groups stratified by risk score, *TP53* mutation status (**C**), *TTN* mutation status (**D**), and *SPOP* mutation status (**E**)

Next, somatic mutation data from the TCGA cohort of PCa patients were obtained, processed, and analyzed by the “maftools” R package. We showed driver genes with mutations in at least 3% of the samples in the two groups. Notably, the high-risk group presented a higher frequency of driver gene mutations (Fig. 10A, B). Considering that *TP53*, *TTN,* and *SPOP* have the highest mutation frequency, the association between mutations in these genes and PCa patient prognosis was then explored. Strikingly, patients with low-risk scores and *TP53* mutations exhibited significantly better outcomes than did those with high-risk scores and *TP53* mutations, while patients with low-risk scores and wild-type *TP53* demonstrated significantly better outcomes than did those with wild-type *TP53* and high-risk scores (*P* < 0.001; Fig. 10C). Interestingly, patients who had *TP53* mutations and low-risk (or high-risk) scores had similar prognoses to patients who had wild-type *TP53* and low-risk (or high-risk) scores. Similar results were found in other driver genes (*P* < 0.001; Fig. 10D, E). The above findings demonstrated that the MTGs-based signature may have greater prognostic significance than the mutation status of *TP53*, *TTN*, or *SPOP*.

### Construction and assessment of a predictive nomogram

**Fig. 11:**
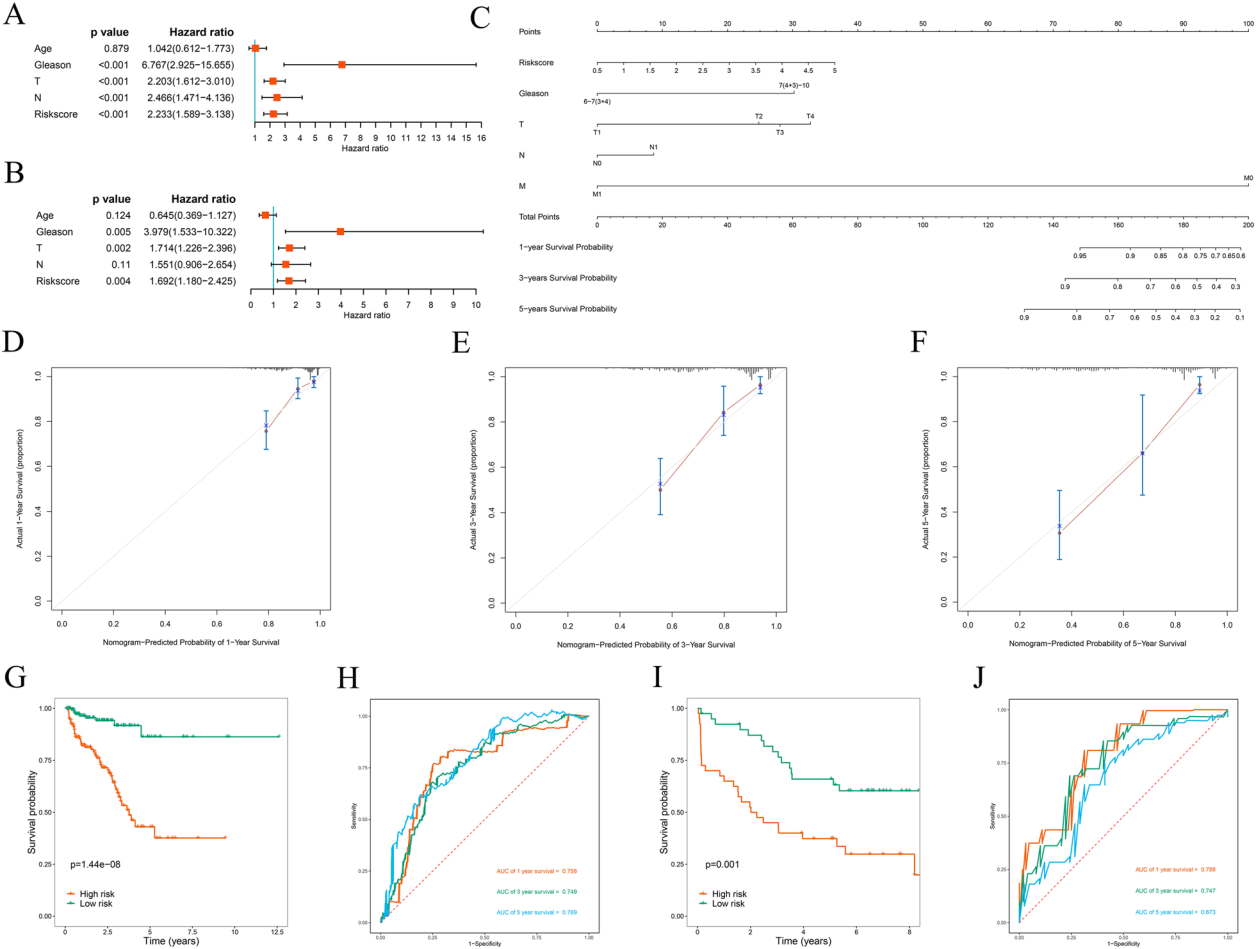
Prognostic significance of different clinical parameters in the TCGA cohort and the construction of a nomogram. **A** Univariate Cox regression analysis of the risk score and clinical parameters. **B** Multivariate Cox regression analysis of the risk score and clinical parameters. **C** Nomogram for predicting the 1-year, 3-year, and 5-year BCRs of PCa patients. **D**, **E**, **F** Calibration curves of the nomogram for predicting BCR at 1, 3, and 5 years. **G** Kaplan–Meier survival curve analysis of BCRs between the high- and low-risk groups in the TCGA cohort based on the nomogram. **H** 1-year, 3-year, and 5-year ROC curves of PCa patients in the TCGA cohort based on the nomogram. **I** Kaplan–Meier survival curve analysis of BCRs between the high- and low-risk groups in the GSE70769 cohort based on the nomogram. **J** 1-year, 3-year, and 5-year ROC curves of PCa patients in the GSE70769 cohort based on the nomogram

Cox regression analyses were performed on common clinical variables and risk scores to explore the connection between the prognostic signatures of PCa patients and outcomes. Univariate Cox regression analysis showed that the Gleason score, risk score, N stage, and T stage were significant prognostic factors associated with BCR (*P* < 0.001, Fig. 11A). while risk score (*P* = 0.004), T stage (*P* = 0.002), and Gleason score (*P* = 0.005) were independent predictive markers connected to BCR according to the multivariate Cox regression (Fig. 11B). Subsequently, a nomogram including common clinical variables and the risk score was constructed to quantitatively forecast BCR rates in PCa patients. The nomogram is shown in Fig. 11C. The calibration curves at various time points revealed that the nomogram has high prediction accuracy (Fig. 11D–F). To further confirm the predictive power of the nomogram, the TCGA and GSE70769 cohorts were used for analysis. The prognosis of PCa patients in the high-risk group exhibited worse in the survival analysis (*P* = 1.44e-08; Fig. 11G). In addition, ROC curve analysis was employed to assess the predictive performance of the nomogram, and the AUCs for BCR at 1 year, 3 years and 5 years were 0.756, 0.749, and 0.789, respectively (Fig. 11H). A similar predictive performance was observed in the GSE70769 cohort according to the nomogram (Fig. 11I, J). The aforementioned findings suggested that the signature-based nomogram showed better predictive performance.

### Expression analysis of prognostic MTGs in PCa

**Fig. 12:**
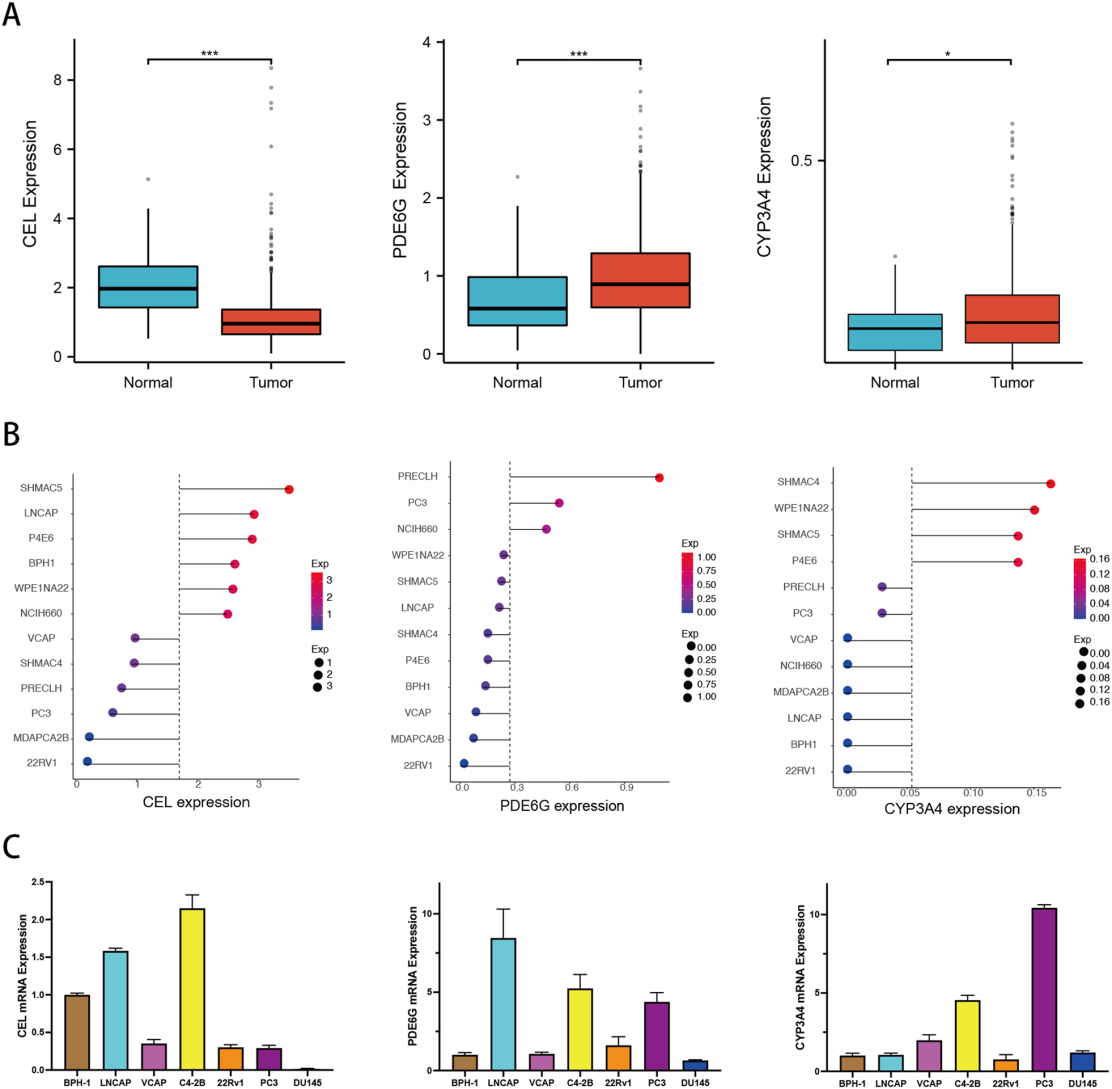
Expression analysis of prognostic MTGs in PCa. **A** The differential expression of *CEL*, *PDE6G*, and *CYP3A4* in PCa tissue from the TCGA database. **B** The relative expression of *CEL*, *PDE6G*, and *CYP3A4* in prostate cell lines from the CCLE database. **C** The relative expression of *CEL*, *PDE6G*, and *CYP3A4* in prostate cell lines was determined via RT‒PCR

It was a good predictive approach for the MTGs-based prognostic signature to identify BCR in PCa patients from the above analysis. Nevertheless, the underlying association between the development of PCa and the expression of prognostic MTGs has not been determined. The comparisons between tumor tissue and normal tissue showed that *PDE6G* and *CYP3A4* expression, but not *CEL* expression, in PCa tissue was considerably higher than that in normal prostate tissue (Fig. 12A). Similarly, gene expression profiling analysis of various prostate cancer cell lines from the CCLE database demonstrated that, compared with that in the benign prostatic hyperplasia cell line BPH-1, *CEL* gene expression was decreased in most prostate cancer cells, while *PDE6G* and *CYP3A4* gene expression was increased (Fig. 12B). Consistently, RT‒PCR revealed similar expression patterns of the *CEL*, *PDE6G*, and *CYP3A4* genes in most PCa cell lines (Fig. 12C). The above results suggested that the differential expression of prognostic MTGs between normal and tumor tissues might play a vital role in PCa development.

## Discussion

Tumor growth and progression are characterized by the intricate interplay between various cell types, forming complex networks that influence each other's functions and metabolic reprogramming [28]. For instance, tumor-surrounding adipocytes can regulate the function of tumor cells and their response to drugs. In PCa, it has been reported that IGF-1 secreted by periprostatic adipose tissue (PPAT) reduces the response of PCa cells to docetaxel through a TUBB2B-dependent mechanism [29]. Similarly, PPAT-released TGF-β enhanced PCa cell migration by upregulating the expression of connective tissue growth factor [30]. The crosstalk between PPAT and PCa cells could affect cancer progression [31]. Additionally, immune cells and metabolic reprogramming play a crucial role in cancer progression [32]. To perform their necessary functions, immune cells can be fine-tuned in the microenvironment for metabolic instrumentalization. For example, macrophages exhibit distinct metabolic pathways during the activation of M1 and M2 macrophages. Specifically, M1 macrophages primarily rely on fatty acid biosynthesis, pentose phosphate pathway, and anaerobic glycolysis to support anabolic processes, while M2 macrophages predominantly utilize oxidative phosphorylation for catabolic processes to maintain their needs [33]. Therefore, understanding how metabolism regulates the differentiation, function, and plasticity of immune cells, as well as how intracellular metabolism affects the function of immune cells, has emerged as the focus of current research. Moreover, despite the good efficacy of certain monoclonal antibodies in some cancer patients, the response rates remain modest and short-lived. This limited response might be attributed to the multiple mechanisms that inhibit antitumor immune function in adverse tumor environments and metabolism [34, 35]. It is recognized that the function of immune cells could be affected by the metabolic changes occurring in cancer cells, which contribute to tumor immune escape, so increasing attention has been directed toward developing immunotherapy strategies that target metabolic pathways [36]. For example, combining IDO inhibitors with checkpoint blockade therapy has shown promising results in clinical trials [37]. It is worth noting that not all combination treatments are better than treatment alone, as a meta-analysis showed that the combination of androgen receptor-axis-targeted (ARAT) agents and docetaxel did not have a significant overall survival benefit compared with ARAT agents [38].

In our study, we investigated metabolic alterations based on the immune background of PCa patients. We identified 48 differentially expressed MTGs between the low-immunity and high-immunity groups. Functional enrichment analysis revealed that these MTGs were involved primarily in amino acid metabolism, glucose metabolism, drug metabolism, lipid synthesis, and the biosynthesis of coenzymes. Bioenergetics and metabolism are the core to meet the multiple nutritional requirements of malignant tumor cells [39]. In this case, fermentation glycolysis or the “Warburg effect”, despite the low production of ATP/glucose molecules, is best suited for the production of anabolic precursors required for the rapid differentiation of embryonic tissue and tumors [40].

Next, we performed Cox and LASSO regression analyses on these MTGs, and three MTGs, namely, *CEL*, *CYP3A4*, and *PDE6G*, were identified as the genes that were most strongly correlated with prognosis. The human carboxyl ester lipase (*CEL*) gene encodes a digestive enzyme that is involved primarily in the hydrolysis and absorption of cholesterol and fat-soluble vitamins [41]. Dalva et al. [42] found that copy number variation and variable-number tandem repeat length polymorphisms in the *CEL* gene were risk factors for pancreatic cancer. And our study showed that the *CEL* gene is expressed at low levels in PCa. *CYP3A4*, a member of the CYP450 family, has been implicated in the occurrence and progression of various diseases. It has been reported that polymorphisms in the *CYP3A4* gene affect the expression level and activity of CYP3A4 and potentially contribute to carcinogenesis [43]. Moreover, CYP3A4 is involved in the synthesis of epoxyeicosatenoic acids (EETs) and facilitates the growth of breast cancer cells via STAT3 activation mediated by EET [44]. In addition, Fujimura et al. [45] found that an increased Gleason score in human PCa was linked to reduced *CYP3A4* expression, and decreased *CYP3A4* expression considerably reduced cancer-specific survival. Consistently, the *CYP3A4* gene was highly expressed in most PCa cell lines, which was demonstrated by analyzing public databases and RT–PCR results. *PDE6G*, a member of the PDE6 family, is a cGMP-specific phosphodiesterase that regulates the specificity of cAMP or cGMP as a substrate [46]. Dong et al. [47] demonstrated that the *PDE6* expression level in human breast cancer cells was abnormal. Similarly, in our investigation, the *PDE6G* gene was indicated to be highly expressed in PCa. The aforementioned findings indicated that the three prognostic MTGs might influence the occurrence and progression of PCa, but their specific mechanisms need further experimental verification.

A prognostic-related signature was then constructed based on the three MTGs to predict BCR in PCa patients. We stratified PCa patients based on different clinical variables, such as the Gleason score, age, M stage, N stage, and T stage, to verify the independent predictive performance of the signature. Further stratified analysis indicated the predictive power of the MTGs-based prognostic signature for BCR in PCa patients, regardless of clinical variables. Our findings demonstrated that the 3-MTGs signature had good accuracy in distinguishing PCa patients with BCR.

miRNAs, which act as oncogenic and tumor suppressor molecules, are essential for tumorigenesis and progression. Studies have shown that many miRNAs exert their effects on diverse metabolic molecules and pathways, which may be the key pathways for energy recombination in tumor cells [48]. Some miRNAs have been found to promote the metabolic reprogramming of tumor cells by responding to metabolic signals, thereby modulating cellular conditions and inducing specific phenotypes [49]. In this study, we identified 9 upstream miRNAs involved in the regulation of prognostic MTGs that may influence the occurrence and progression of PCa, but further experiments are warranted. Additionally, our study revealed that the expression levels of common ICI genes were significantly different between the signature-based low- and high-risk groups. Moreover, we analyzed the difference in immunotherapy response between the two groups based on the TIDE algorithm. TIDE prediction score, as an immunotherapy prediction algorithm, has been widely used, and its predictive function has been successfully verified [21]. Collectively, these findings suggested that our signature holds promise as a reliable immune biomarker for tumor therapy.

In addition to the methods used above, an artificial neural network (ANN) model is also another useful approach to identify and validate PCa biomarkers, which facilitate personalized management of PCa [50]. For example, the ANN model based on clinical tests such as the prostate health index, Proclarix blood test, or multiparametric magnetic resonance had high accuracy in the recognition of progressive PCa at initial diagnosis [51, 52]. Therefore, the ANN model combining the three prognostic MTGs and PCa biomarkers in clinical tests may identify new tools with increased accuracy for personalized management of PCa.

Overall, our MTGs-based signature offers a novel quantitative approach for the prediction of PCa patient prognosis. The results also provide valuable insights for further investigations into the mechanisms and processes underlying metabolic alterations in the immune microenvironment of PCa. However, there are several limitations to our study. First, to validate our research, our model needs prospective cohorts and more external data. Second, the specific roles of the three prognostic MTGs and the underlying mechanisms require further experimental exploration.

## Conclusions

In this study, based on the immune background of PCa patients, we comprehensively explored the cytological functions and biological mechanisms of MTGs and constructed a prognostic-related signature that could independently predict BCR in PCa patients. Our study provides clues for the prediction of PCa patients prognosis and may help elucidate metabolic processes and mechanisms within the immune microenvironment of PCa. Moreover, the predictive signature demonstrated high sensitivity in identifying PCa patients who are likely to have a favorable response to immunotherapy.

### Supplementary Information

**Additional file 1: Figure S1.** LASSO regression analysis for screening prognosis-related MTGs.

**Additional file 2: Table S1.** miRNA and metabolic genes regulatory networks. **Table S2.** The primer sequences of prognostic MTGs.

## Data Availability

The data and materials can be obtained by contacting the corresponding author.

## Abbreviations

TME: Tumor microenvironment
TILs: Tumor-infiltrating lymphocytes
PCa: Prostate cancer
ADT: Androgen deprivation therapy
TCGA: The Cancer Genome Atlas
TIME: Tumor immune microenvironment
BCR: Biochemical relapse
Foxp3 +: Forkhead box P3
Tregs: Regulatory T cells
GEO: Gene expression omnibus
RMA: Robust multi-array analysis
SsGSEA: Single‐sample gene sets enrichment analysis
ESTIMATE: Estimation of stromal and immune cells in tumor tissues
CIBERSORT: Cell type identification by estimating relative subsets of RNA transcripts
MTGs: Metabolic genes
KEGG: Kyoto encyclopedia of genes and genomes
GSEA: Gene set enrichment analysis
FC: Fold change
FDR: False discovery rate
LASSO: Least absolute shrinkage and selection operator
ROC: Receiver operating characteristic
GO: Gene ontology
AUC: Area under the receiver operating characteristic curve
ICI: Immune checkpoint inhibitor
PCA: Principal component analysis
