# Supplementary figures and images for: Identification and validation of an individualized metabolic prognostic signature for predicting the biochemical recurrence of prostate cancer based on the immune microenvironment
Source: Eur J Med Res. 2024 Jan 31;29:92. doi: 10.1186/s40001-024-01672-3 (PMC10829481; doi:10.1186/s40001-024-01672-3)

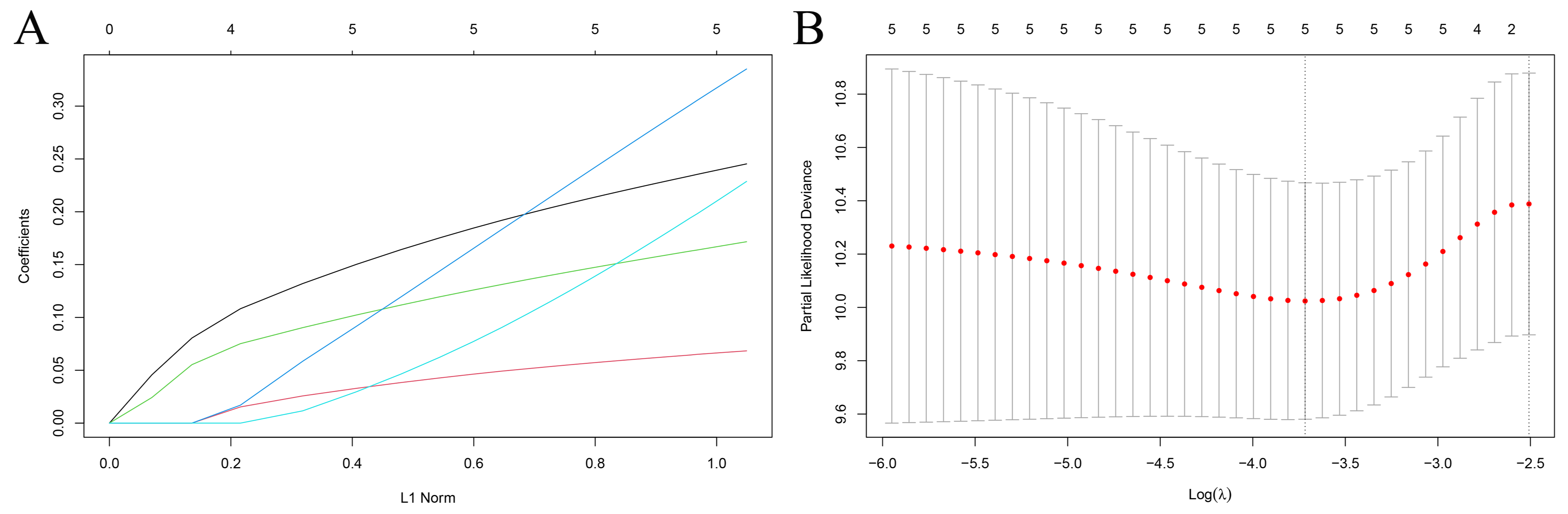

Supplement: Supplementary file 1 — Additional file 1: Figure S1. LASSO regression analysis for screening prognosis-related MTGs. [file 40001_2024_1672_MOESM1_ESM.tif]
