# Supplementary material for: Identification and validation of an individualized metabolic prognostic signature for predicting the biochemical recurrence of prostate cancer based on the immune microenvironment
Source: Eur J Med Res. 2024 Jan 31;29:92. doi: 10.1186/s40001-024-01672-3 (PMC10829481; doi:10.1186/s40001-024-01672-3)
Supplement: Supplementary file 2 — Additional file 2: Table S1. miRNA and metabolic genes regulatory networks. Table S2. The primer sequences of prognostic MTGs. [file 40001_2024_1672_MOESM2_ESM.docx]

Table S1. MiRNAs and metabolic genes regulatory networks.

| miRNAs | Metabolic genes | Coefficient | P value | Regulation |
| --- | --- | --- | --- | --- |
| Has-miR-1224-5p | CEL | 0.479 | 1.01E-29 | Positive |
| Has-miR-184 | CEL | 0.529 | 6.38E-37 | Positive |
| Has-miR-592 | CEL | 0.662 | 1.05E-63 | Positive |
| Has-miR-1224-5p | CYP3A4 | 0.624 | 1.20E-54 | Positive |
| Has-miR-184 | CYP3A4 | 0.705 | 2.04E-75 | Positive |
| Has-miR-190b-5p | CYP3A4 | 0.338 | 1.08E-14 | Positive |
| Has-miR-592 | CYP3A4 | 0.917 | 5.02E-198 | Positive |
| Has-miR-146a-3p | PDE6G | 0.372 | 1.06E-17 | Positive |
| Has-miR-146b-5p | PDE6G | 0.352 | 7.29E-16 | Positive |
| Has-miR-3614-3p | PDE6G | 0.305 | 3.95E-12 | Positive |
| Has-miR-3614-5p | PDE6G | 0.311 | 1.46E-12 | Positive |
| Has-miR-7702 | PDE6G | 0.300 | 9.10E-12 | Positive |

Table S2. The primer sequences of prognostic MTGs

| (human) | Forward 5'-3' | Reverse 5'-3' |
| --- | --- | --- |
| GAPDH | TGGTCACCAGGGCTGCTTTTA | CATCGCCCCACTTGATTTTG |
| CEL | GTCACCTTCAACTACCGTGTC | GGCCGCGATATTCCTCTTCAC |
| PDE6G | GTTCAAGGGTTTGGGGACGA | GGTTTAGAGCACAGTGGGCA |
| CYP3A4 | TGCATTGGCATGAGGTTTGC | ACTTACGGTGCCATCCCTTG |
